# Loss of Prefrontal Cortical Higher Cognition with Uncontrollable Stress: Molecular Mechanisms, Changes with Age, and Relevance to Treatment

**DOI:** 10.3390/brainsci9050113

**Published:** 2019-05-17

**Authors:** Dibyadeep Datta, Amy F. T. Arnsten

**Affiliations:** Department Neuroscience, Yale Medical School, New Haven, CT 06510, USA; Dibyadeep.datta@yale.edu

**Keywords:** prefrontal cortex, stress adolescence, aging, calcium, cAMP, dopamine, norepinephrine

## Abstract

The newly evolved prefrontal cortex (PFC) generates goals for “top-down” control of behavior, thought, and emotion. However, these circuits are especially vulnerable to uncontrollable stress, with powerful, intracellular mechanisms that rapidly take the PFC “off-line.” High levels of norepinephrine and dopamine released during stress engage α1-AR and D1R, which activate feedforward calcium-cAMP signaling pathways that open nearby potassium channels to weaken connectivity and reduce PFC cell firing. Sustained weakening with chronic stress leads to atrophy of dendrites and spines. Understanding these signaling events helps to explain the increased susceptibility of the PFC to stress pathology during adolescence, when dopamine expression is increased in the PFC, and with advanced age, when the molecular “brakes” on stress signaling are diminished by loss of phosphodiesterases. These mechanisms have also led to pharmacological treatments for stress-related disorders, including guanfacine treatment of childhood trauma, and prazosin treatment of veterans and civilians with post-traumatic stress disorder.

## 1. Introduction

The prefrontal cortex (PFC) provides “top-down” control of behavior, thought, and emotion. However, these newly evolved circuits are especially vulnerable to uncontrollable stress, with built-in mechanisms to rapidly take the PFC “off-line” and switch the brain from a reflective to reflexive state. The current review summarizes the role of PFC circuits in top-down control, the unique molecular mechanisms governing PFC synapses that induce this rapid loss of function during stress exposure, and, with repeated stress, the atrophy of dendrites and spines. Understanding the molecular events that drive these powerful changes in brain state has direct relevance to the etiology of stress-related disorders such as depression, post-traumatic stress disorder (PTSD), substance abuse, schizophrenia, and late onset Alzheimer’s disease (LOAD). These molecular mechanisms also help to explain why the PFC is so susceptible to stress pathology during adolescence (when catecholamine expression is increased in the PFC), and with advanced age (when the molecular “brakes” on stress signaling are diminished). Finally, understanding the molecular basis of the stress response in PFC has led to pharmacological treatments that are in widespread clinical use, a rare instance of successful translation from animals to humans.

## 2. The PFC Circuitry in Primates Serving Top-Down Control

The PFC provides top-down regulation of thought, action, and emotion [1], and has extensive connections to either promote or inhibit these neural events [2,3,4,5,6]. The PFC expands greatly in primate evolution, with the ventral and medial PFC (vmPFC) specialized for the regulation of emotion (internal states), while the more dorsal and lateral regions of the PFC (dlPFC) mediate cognition (external states) [2,3,4,7]. The most rostral, frontal pole serves metacognition, e.g., insight about oneself and others [8]. These regions all interconnect to provide a holistic mental state, where more newly evolved rostral and dorsolateral regions can provide top-down regulation of more ancient, caudal structures.

Evidence of “top-down” regulation by rostral and dlPFC–physiological recordings from monkeys have provided extensive evidence of “top-down” regulation by the dlPFC and rostral PFC circuits. Early studies found that these areas have the ability to generate neural activity in the absence of sensory stimulation, e.g., during a working memory task, the foundation of abstract thought [9,10]. This persistent firing during working memory could also be used to guide behavior, e.g., inhibiting an inappropriate response [11], and to suppress responding to distractors [12]. More recent studies have shown extensive roles in categorization and abstract rules, top-down control of attention, and strategic decision-making, e.g., References [12,13,14,15,16,17]. Recordings from rostral medial PFC circuits have revealed signatures of high order cognitive capabilities, including social aspects of decision-making such as “theory of mind,” i.e., understanding the minds of others [18], as well as metacognitive self-evaluation, i.e., insights about one’s own decisions, in the frontal pole (area 10) [19,20]. These physiological recordings are consonant with studies of monkeys and patients with lesions to these regions, which demonstrate deficits in the top-down control of social and emotional behavior (reviewed in Reference [21]).

**Figure 1:**
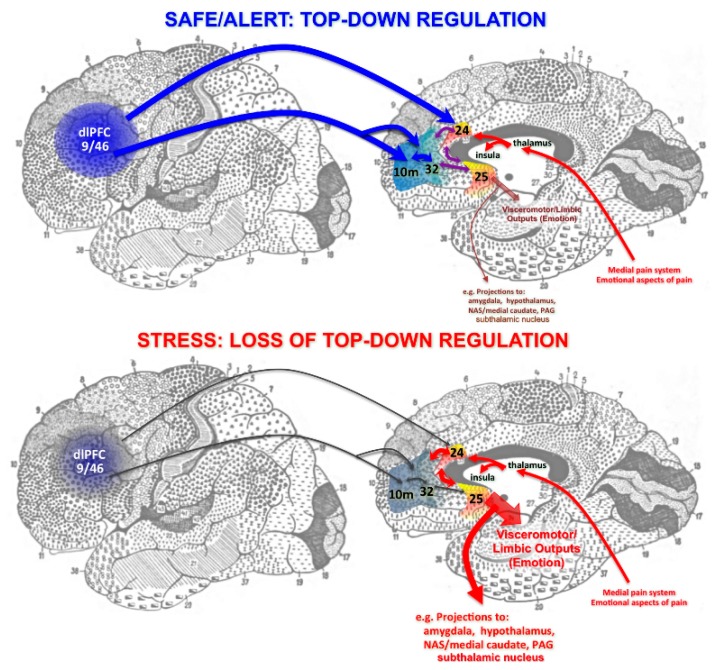
Schematic diagram of prefrontal cortex (PFC) circuits providing top-down regulation of emotion, and the effects of arousal state on connectivity. (**Top**): Under non-stress conditions, newly evolved dorsolateral PFC (dlPFC) and rostral areas (e.g., BA10) project back to anterior (BA24) and subgenual (BA25) cingulate via BA32 to regulate visceromotor output and emotional response. Note that these cingulate areas are part of the pathway that mediates the emotional aspects of the pain response. (**Bottom**): Under conditions of uncontrollable stress, the connectivity of dlPFC and the rostral aspect of medial PFC are weakened, and the top-down suppression of BA25 is diminished, promoting activation of subcortical structures such as the amygdala. Anatomical projections are based on tracing studies in monkeys [38], but are portrayed on a Brodmann drawing of the human brain to facilitate translation to human brain imaging results.

Ventral and medial PFC circuits regulate emotion. The ventral (orbital) and medial PFC provide flexible evaluation of affective information such as reward and punishment [18,22,23,24,25]. Although these regions are often referred to in a unitary fashion as simply “vmPFC,” a more careful examination of the human imaging data, coupled with the known anatomical connections of this region in nonhuman primates, indicates important differentiations which likely mediate distinct functional contributions. The medial PFC includes the cingulate cortices: the anterior cingulate cortex, also known as Brodmann Area (BA)24, and the cingulate cortex under the genu of the corpus callosum, often called the subgenual cingulate, or BA25 (Figure 1; numbering scheme of Reference [26]). BA24 and BA25 as well as the insular cortex are all key parts of a medial circuit that processes the emotional aspects of pain [27,28]. These structures are overactive in neuropathic pain [29], and have been surgically removed to treat intractable pain [30]. The anterior cingulate also activates with cognitive conflict, e.g., mental errors [31], emphasizing the mental nature of its function. The anterior cingulate projects to nearby premotor areas, e.g., BA6d, to influence motor responses such as eye or hand movements (Figure 1). Information also flows from BA24 and the insular cortex to BA25, which serves as the major visceromotor output for the PFC (Figure 1). BA25 is of particular interest given its overactivity in depression, and is thus a focus of deep brain stimulation treatment [32]. This area has extensive projections to limbic areas such as the amygdala, ventral striatum, and hypothalamus to control emotion and visceral responses [3,33]. This includes projections to hypothalamus and brainstem centers that coordinate the stress response [34], consistent with its activity correlating with increased cortisol release in stressed human subjects (see Table S2 in the Supplement of Reference [35]). BA25 also interconnects with the medial subthalamic nucleus [36], which, if playing a role similar to that in motor circuits, may provide pervasive inhibition relevant to symptoms of “mental paralysis,” a hypothesis supported by the antidepressant effects of subthalamic deep brain stimulation [37]. 

Anatomical tracing studies in monkeys [38] indicate that dlPFC may be able to regulate BA25 through indirect connections via areas BA10m and BA32 (Figure 1A). Human imaging studies suggest these connections may be important in regulating stress and depression, as dlPFC functional connectivity correlates with that of vmPFC BA32 as subjects overcome their response to stress [35], and as activity in this “medial corridor” is related to a sustained anti-depressant response to deep brain stimulation [39]. Furthermore, the antidepressant effects of TMS to strengthen the left dlPFC have been related to its ability to reduce the activity of BA25 [40], supporting the circuit model shown in Figure 1. Conversely, dlPFC and medial PFC deactivate during uncontrollable stress (Figure 1B), as described below. The loss of top-down control by the dlPFC and rostral circuits with stress has been of special interest, as these newly evolved circuits are especially vulnerable in neuropsychiatric disorders. Thus, they have been a focus of neurobiological research.

## 3. The Microcircuitry for Generating Top-Down Goals for Regulating Thought, Action, and Emotion

**Figure 2:**
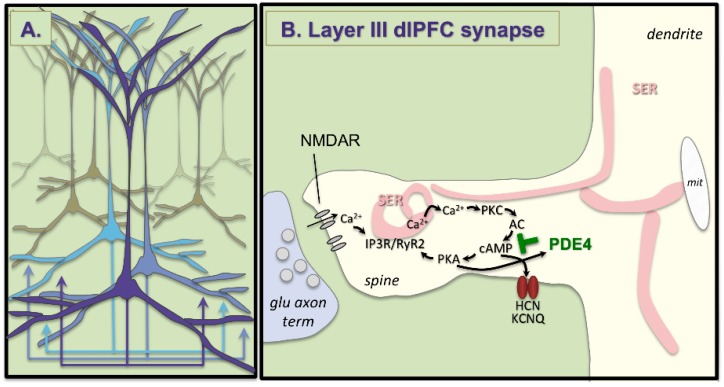
Recurrent excitation in deep layer III of the dlPFC. (**A**) Schematic drawing of clusters of pyramidal cells in deep layer III of dlPFC with shared characteristics that excite each other through extensive recurrent excitatory glutamatergic connections to keep information “in mind” in the absence of sensory stimulation. (**B**) A glutamate synapse on a pyramidal cell spine in deep layer III of dlPFC. These synapses depend on NMDAR stimulation, and have extensive elaboration of the calcium-containing smooth endoplasmic reticulum (SER) “spine apparatus” in the spine, where there is evidence of feedforward calcium-cAMP signaling, which can open HCN and KCNQ channels to reduce firing. This process is held in check by the phosphodiesterases (PDE4), which catabolize cAMP and are anchored to the spine apparatus by DISC1 [56].

The work of Goldman-Rakic [41] and of González-Burgos [42,43] has uncovered the microcircuitry in deep layer III of dlPFC that allows the persistent representation of information in the absence of sensory stimulation, the neural basis for top-down control. Tract tracing studies of primate dlPFC in vivo [41] and in slices [42] have shown extensive horizontal connections in deep layer III, the anatomical basis for extensive recurrent excitation. This research showed that the persistent firing of neurons across the delay period in a working memory task arises from the recurrent excitation of pyramidal cells with shared characteristics (Figure 2A). For example, a group of pyramidal cells will continue to fire across the delay epoch after a spatial cue is presented at a location 90° from the fixation point, but not other locations. These neurons are able to continue firing without sensory stimulation due to their recurrent excitatory network connections, as illustrated in Figure 2A. These pyramidal cells excite each other through glutamatergic NMDAR synapses even after the cue has been extinguished, thus maintaining the memory of 90° across the delay period. Conversely, the spatial tuning of neuronal firing is refined by lateral inhibition from parvalbumin-containing GABA interneurons, reducing firing for nonpreferred information [41]. This contrasts with classic circuits in the sensory cortex, where feedforward inhibition rather than lateral inhibition is the rule. Studies of dlPFC slices were able to identify GABAergic basket and chandelier cells inhibiting pyramidal cells to produce lateral inhibition, consistent with the in vivo recordings [43]. These layer III microcircuits have expanded greatly in primate brain evolution [44], and are the neurons most afflicted in schizophrenia, with loss of dendrites and spines from pyramidal cells, and compensatory weakening of GABA interneurons [45]. These pyramidal cells also fill with neurofibrillary tangles and degenerate in Alzheimer’s Disease [46]. It is not known whether medial and rostral PFC regions also contain recurrent microcircuits, which would be an important area for future research. 

## 4. The Unique Neurotransmission and Neuromodulation of dlPFC Synapses

The recurrent excitatory synapses on pyramidal cell spines in deep layer III of dlPFC have characteristics that render them especially vulnerable to stress and atrophy, including important roles of calcium signaling (Figure 2B). These characteristics include unusual glutamate neurotransmission and neuromodulatory actions, where cAMP-calcium signaling weakens rather than strengthens network connectivity, e.g., during uncontrollable stress exposure.

Neurotransmission classic glutamatergic circuits, e.g., in V1, rely heavily on AMPA receptor (AMPAR) stimulation, which provides the permissive excitation for NMDA receptor (NMDAR actions during neuroplasticity [47]. These circuits have few NMDAR with NR2B subunits in the adult, although they predominate early in development [48]. In contrast, dlPFC delay cells have only a minor reliance on AMPAR, and are greatly reliant on NMDAR-NR2B, which are found exclusively in the post-synaptic density and not at extra-synaptic locations [49], and are concentrated in pyramidal cell synapses [50]. Layer III reliance on NMDAR is also seen in human dlPFC, where pyramidal cells express a greater NMDAR than AMPAR message, while the converse is true in layer V [51]. Layer III delay cells also do not rely on AMPAR to permit NMDAR actions. Instead this permissive role is played by cholinergic stimulation [52], which occurs during waking but not deep sleep, contributing to conscious cortical activity. The reliance of layer III dlPFC circuits on NMDAR-NR2B is particularly interesting, as these receptors close slowly and allow a large amount of calcium into the post-synaptic spine (schematically shown in Figure 2B), and calcium plays a major neuromodulatory role in determining the strength of these network connections through powerful neuromodulatory actions. 

Neuromodulation layer III dlPFC pyramidal cells also have unique neuromodulatory influences, where calcium-cAMP signaling weakens connections by opening potassium (K^+^) channels on spines. We have proposed that calcium plays a critical, negative feedback role in these recurrent excitatory circuits where there is little feedback inhibition, and thus may prevent excessive neuronal firing [6]. We see evidence of feedforward calcium-cAMP signaling in spines near K^+^ channels that are regulated by calcium itself (e.g., SK channels [53]), or by cAMP-PKA signaling (HCN and KCNQ channels) [6,54,55]. As schematized in Figure 2B, layer III dlPFC spines have extensive smooth endoplasmic reticulum (SER), which stores and releases calcium into the cytosol (the SER is called the spine apparatus where it extends and elaborates in the spine). In layer III of dlPFC, the spine apparatus is the focus of extensive cAMP-signaling machinery [54,56,57,58], consistent with feedforward calcium-cAMP signaling. Thus, calcium release can drive the production of cAMP, which activates PKA, which drives further calcium release [55]. Feedforward signaling promotes a rapid build-up of calcium-cAMP-PKA activity to open K^+^ channels and reduce firing. These detrimental actions are prevented under optimal arousal conditions by NE stimulation of α2A-AR [54] and NAAG/glutamate stimulation of mGluR3 [59]. These receptors are concentrated on layer III dlPFC post-synaptic spines where they inhibit cAMP signaling, close K^+^ channels, and enhance firing. Under healthy conditions, PKA also activates the phosphodiesterases (e.g., PDE4A) to catabolize cAMP and thus provide brakes on cAMP-calcium signaling after it has been activated. However, this negative feedback is lost with age and with inflammation, as described below. 

## 5. Stress Rapidly Takes PFC Circuits “Offline”

**Figure 3:**
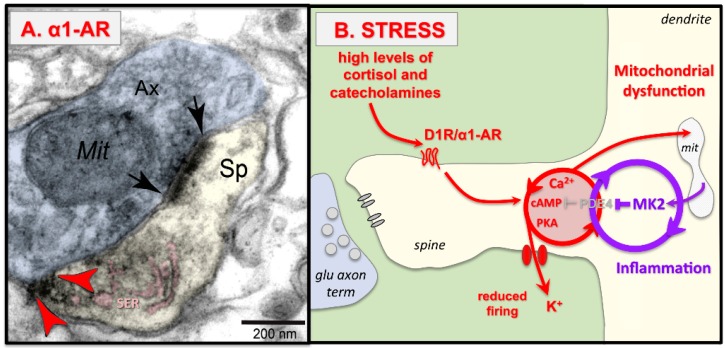
Uncontrollable stress weakens PFC synaptic connectivity through α1-AR and D1R drive of calcium-cAMP-K^+^ signaling. (**A**) DAB immunolabeling shows that α1-AR (red arrowheads) are localized on dendritic spines in layer III dlPFC near the calcium-containing SER spine apparatus, pseudocolored in pink. The synapse is indicated by black arrows. Sp = spine, Ax = axon terminal, Mit = mitochondrion. Figure 3A adapted from Reference [70]. (**B**) Schematic drawing showing that uncontrollable stress induces cortisol and catecholamine release; high levels of NE and DA activate α1-AR and D1R, which drive feedforward, calcium-cAMP opening of HCN and KCNQ channels to reduce cell firing. With sustained stress exposure, calcium overload of mitochondria may induce inflammatory responses such as MK2 signaling, which inhibits PDE4 and thus removes the “brakes” on stress signaling pathways, ultimately leading to spine loss.

Even mild uncontrollable stress increases catecholamine release in the PFC to drive feedforward calcium-cAMP-K^+^ signaling and rapidly take the PFC “off-line.” These effects were initially observed in rodent medial PFC, but similar mechanisms have been documented in the primate dlPFC. Due to the early work of Steven Maier, it has long been appreciated that it is the uncontrollable aspect of the stressor that initiates the stress response and leads to cognitive deficits, e.g., Reference [60], and an acute, uncontrollable stress can induce a distracted behavior profile [61]. In contrast, a controllable stressor does not induce dopamine release in the PFC [62]. Biochemical studies documented increased catecholamine release in the rat medial PFC in response to mild uncontrollable stress [63,64,65]. High levels of dopamine (DA) D1R and norepinephrine (NE) α1-AR stimulation in the PFC impair working memory performance by driving feedforward calcium-cAMP-K^+^ signaling [66,67,68,69], as schematically illustrated in Figure 3. Both α1R (Figure 3A; [70]) and D1R [57,71] have been localized on dendritic spines in layer III of primate dlPFC, and their activation drives feedforward calcium-cAMP signaling (Figure 3B). Thus, stimulation of α1R or D1R reduces the task-related firing of dlPFC neurons in monkeys performing a working memory task, while blockade or closure of HCN channels rescues firing [68,69,70,72]. These stress signaling pathways may interact with neuroinflammation, which may remove the brakes on the stress response by inhibiting PDE4s, as illustrated in Figure 3B. Specifically, in vitro studies have shown that MK2 inflammatory signaling can inhibit PDE4 regulation of the stress response by un-anchoring PDE4s from their correct location and preventing PKA activation of PDE4 negative feedback on the cAMP-PKA response [73,74]. Similar actions in PFC pyramidal cells would accelerate and prolong the response to uncontrollable stress. 

In contrast to the PFC, high levels of catecholamine strengthen the emotional responses of the amygdala and the habitual responding of the striatum [75,76,77], and can enhance the functioning of the primary sensory cortex [78]. Thus, high levels of catecholamine released during uncontrollable stress switch the brain from a slow, thoughtful, reflective PFC-regulated state to a more reactive, reflexive state that may be advantageous during danger, but would be detrimental when more thoughtful solutions are needed [79]. 

The detrimental effects of stress-induced catecholamine release are exacerbated by glucocorticoids (cortisol in primates, corticosterone in rodents). Cortisol blocks extraneuronal catecholamine transporters, and thus expands the effects of catecholamines [80]. Corticosterone has been shown to exacerbate the catecholamine response in rat brain, both in impairing PFC working memory [81] and in fortifying the amygdala’s enhanced consolidation of emotional memories [82]. 

Stress-induced PFC dysfunction has now been documented in rats, monkeys, and humans, indicating that this is a highly conserved response. For example, exposure to violent images impairs performance of a working memory task and reduces the activity of the dlPFC in humans [83], and the degree of impairment is related to COMT genotype, with greater catecholamines associated with greater dlPFC dysfunction [84]. Functional brain imaging has also been used to assess the dynamic changes in PFC and cortisol release in response to viewing violent images. This study showed that cortisol release correlated with activation of a caudal region that included BA25, while the suppression of the cortisol response was related to activity in the vmPFC (e.g., BA32), which initially deactivated with stress but reactivated in correlation with coping [35]. Importantly, the reactivation of BA32 correlated with its connectivity with dlPFC, indicating a network of PFC subregions regulating the stress response in humans. It would be helpful to extend this approach to the nonhuman primate, for example to determine the molecular mechanisms governing medial PFC (BA32, BA24, BA25) connectivity and neuronal firing.

## 6. Architectural Changes with Chronic Stress

Chronic stress exposure leads to additional architectural changes, including spine loss and dendritic atrophy from the medial PFC in rodents, and PFC gray matter loss seen with structural imaging in humans. Dendritic atrophy induced by chronic stress was originally observed in the rat hippocampus [85], but has been found to be even more sensitive in the rat medial PFC [86,87,88,89,90]. Dendritic changes are circuit-specific, where PFC pyramidal cells projecting to entorhinal cortex atrophy with chronic stress, while those projecting to and activating the basolateral amygdala show dendritic expansion [89]. The dendrites of amygdala neurons also expand [91], thus strengthening more primitive emotional circuits in concert with the loss of PFC cortical–cortical connections. Importantly, the loss of spines and dendrites in medial PFC correlates with impaired PFC cognitive functioning on working memory [92,93] and attention-shifting [87] tasks, demonstrating that these architectural changes have great functional relevance. Dendritic integrity is restored with a prolonged period of non-stress following the chronic stress, at least in young animals [94]. Antidepressant treatments also induce spinogenesis in medial PFC [90], and longitudinal in vivo imaging has revealed the retraction and subsequent recovery of spines with antidepressant treatment, (although the return of PFC spines was needed for the long-term maintenance of antidepressant effects on motivated escape behavior but not for their initial induction [95]).

Parallel findings have been seen in human subjects with brain imaging, where exposure to repeated stressors is associated with reduced gray matter in the rostral PFC areas that provide top-down control [96]. Sustained stress also has been shown to induce weaker functional connectivity with the dlPFC that correlates with impaired set-shifting attentional regulation that returns to normal after the stress is over [97]. Reduced functional connectivity with PFC is also seen in human subjects with severe childhood abuse [98]. Thus, there are strong parallels between animal and human studies.

It is important to understand the molecular signaling events that cause loss of PFC connections so that we can develop informed strategies for treatment. The loss of dendritic spines with chronic stress can be prevented by daily treatment with agents such as guanfacine that inhibit PKA signaling [93], or chelerythrine, which inhibits PKC signaling [92]. Sustained high levels of feedforward, calcium-PKC cAMP-PKA signaling may cause dendritic atrophy through a variety of downstream mechanisms. For example, excessive calcium leak from the SER can cause calcium overload of mitochondria, initiating inflammatory cascades, and sustained high levels of PKC activity. High levels of PKC activity can phosphorylate MARCKS (myristoylated alanine-rich C-kinase substrate), which detaches the actin cytoskeleton from the plasma membrane, causing spine collapse [99]. It is also noteworthy that PKC activates GSK3β signaling, and both PKC and GSK3β are inhibited by anti-manic medications that rescue PFC gray matter in patients [100,101]. Interestingly, the rapidly-acting antidepressant, ketamine, induces spine formation through activation of mTOR and inhibition of GSK3β signaling [102]. Thus, this is an exciting arena of current research with immediate clinical relevance. 

## 7. Females Have a Greater Stress Response Than Males 

Understanding the molecular basis of the stress response in PFC may help to explain the prominent sex differences in the stress response in animals and humans. Female rats with circulating estrogen have a greater stress response, e.g., due to greater promotion of noradrenergic [103], and dopaminergic [104] actions. A parallel relationship can be seen in humans, where women have reduced expression of COMT [105], and thus less catabolism of catecholamines. Female rats with circulating estrogen have greater stress-induced PFC dysfunction than males (but outperform males when they are ovariectomized) [106,107]. Female rats with circulating estrogen also have a greater architectural response to chronic stress, with increased dendritic expansion in PFC neurons projecting to amygdala [108].

These neurobiological findings may help to explain the increased prevalence of depression and PTSD in women [109,110,111,112]. However, cultural factors also likely play an important role, as women are often given less control in society, and are even encouraged to be helpless, factors that would increase the stress response.

## 8. Increased Susceptibility to Stress-Induced PFC Dysfunction During Adolescence

The neurobiology of stress can also help to explain differences in top-down control over the lifespan. Adolescence is a time of biological susceptibility, for example due to hormonal changes and to pruning of dendritic spines in cortex [113]. Adolescents are especially vulnerable to emotional stress [114,115], with an increased risk of issues such as addiction [116]. Adolescence is also a time of increased DA signaling in the PFC. There is an increased DA innervation of layer III in the macaque dlPFC during adolescence [117,118], and increased expression of D1R on rat prelimbic PFC neurons that project to the nucleus accumbens [119]. Thus, the stress response in PFC may be magnified during adolescence, and may lower the threshold for high-risk behaviors and poor decision-making under emotionally stressful conditions. 

## 9. Increased Vulnerability During Aging Loss of Brakes on Stress Signaling Pathways

The stress response is also magnified with advanced age, due, at least in part, to the loss of regulation at the intracellular and circuit levels. The PFC atrophies with advancing age, with loss of spines from the layer III microcircuits that generate working memory [120]. For example, cortisol levels are higher in elderly individuals, especially in older women [121], and this may involve weaker PFC inhibition of the HPA axis with age. Aging may also alter the stress response through molecular changes. Although there is a decline in PFC DA with advancing age [122], there is also a loss of PDE4 expression from spines, the enzymes that normally catabolize cAMP and hold stress signaling events in check [58]. These data suggest that there may be a higher threshold to activate the stress response in aged individuals, but that once stress signaling is initiated, it would be more prolonged. Increased stress calcium-cAMP signaling in the association cortex may have many consequences that would increase risk of pathology, including mitochondrial abnormalities [123,124], spine loss [120], and phosphorylation of tau [58,125], all of which are seen in layer III of aged monkey dlPFC. Stress is now recognized as a risk factor for late onset AD, with stressful events linked to higher disease onset decades later [126,127,128]. Indeed, recent evidence shows that increased cortisol is a risk factor for disease [129]. The more prominent stress response in women may also help to explain the increased prevalence of late onset AD in women compared to men [130,131], especially as AD pathology begins decades before disease onset, at a time when estrogen mechanisms could exacerbate the biological response to stress.

## 10. Successful Translation to Clinical Treatments

Pharmacological manipulations can protect dlPFC connectivity by inhibiting stress-induced calcium-cAMP-K^+^ signaling and maintaining synaptic efficacy. It is possible that treatments such as GCPII inhibitors that enhance stimulation of mGluR3 may be helpful in the future. However, two treatments that have been helpful in animals—the α2A-AR agonist guanfacine, and the α1-AR antagonist prazosin—are now in widespread clinical use for treating stress-related disorders.

**Figure 4:**
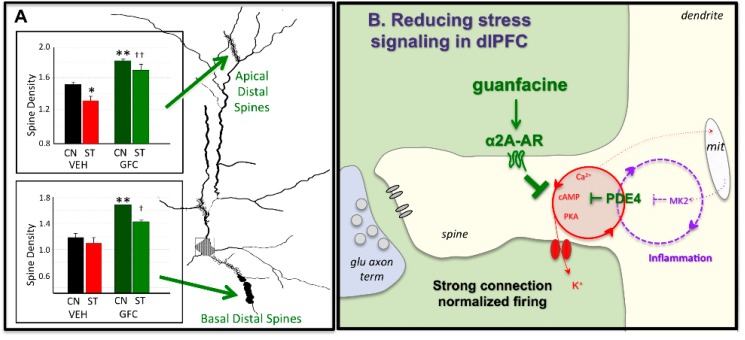
The α2A-AR agonist guanfacine can strengthen PFC connectivity and protect the PFC from stress. (**A**) Chronic restraint stress causes loss of apical distal spines from layer II/III prelimbic PFC pyramidal cells in rats. Daily pre-treatment with guanfacine prevents spine loss and protects working memory function. CN = control, ST = chronic restraint stress, VEH = vehicle, GFC = guanfacine. * or ** significantly different from vehicle control; † or †† significantly different from vehicle stress at *p* < 0.05 or 0.01 levels, respectively. Figure 4A adapted from [93]. (**B**) Schematic diagram showing that guanfacine stimulation of α2A-AR on spines strengthens connectivity and protects PFC from stress by inhibiting cAMP-calcium-K^+^ channel signaling.

The α2A-AR agonist, guanfacine, prevents PFC dysfunction caused by either acute [132] or chronic [93] stress, including rescuing spine loss from PFC neurons (Figure 4A). Studies in monkeys have shown that guanfacine acts by inhibiting cAMP-opening of HCN channels on spines (Figure 4B), strengthening connectivity, persistent firing, and working memory abilities [54]. α2A-AR stimulation also has anti-inflammatory actions, e.g., deactivation of microglia [133]. Based on research in animals, extended release guanfacine (Intuniv^®^, Shire Takada Pharmaceuticals) is now in widespread use for treating ADHD, but is also being used to treat traumatized children [134], including those with oppositional behaviors often arising from maltreatment [135]. 

The α1-AR antagonist prazosin is in widespread use for treating PTSD in adults [136]. As described above, stimulation of α1-AR is a key part of stress-induced PFC dysfunction, and also contributes to the strengthening of amygdala during conditions of high NE release. Prazosin has been found to be helpful in treating combat-related PTSD, including daytime hyperarousal symptoms and improving global clinical status [137]. It is noteworthy that the hyperarousal subscale used to rate PTSD symptoms includes many PFC-related deficits (e.g., impaired concentration, impaired regulation of mood and aggression), in addition to alterations in sleep–wakefulness. Another double-blind placebo-controlled study of civilians addressed whether daytime-only prazosin treatment reduced PTSD symptoms during a trauma-relevant stress paradigm that measured PFC-related executive function through use of an emotional version of the Stroop interference task [138]. Prazosin simultaneously reduced subjective stress and improved cognitive performance [138]. High doses of prazosin may also be helpful in the treatment of daytime PTSD symptoms, when levels of NE release are higher [139]. Prazosin may also be helpful in reducing substance abuse, which is common in PTSD. Initial trials suggest that prazosin can reduce cravings for and use of alcohol in patients with PTSD [140], as well as reducing stress-induced craving for alcohol in subjects without PTSD [141].

## 11. Outstanding Questions and Future Directions

Although there has been remarkable progress in this field, with many similarities bridging across rodent and both nonhuman and human primate species, there are still many outstanding questions. Greater understanding of circuit specific changes with stress exposure is an important arena for future research, but challenging to extend from rodent to primate. The rodent medial PFC represents many primordial features of the PFC, and projections are organized in gradients rather than in discrete subregions [142]. The great expansion of the PFC in primates suggests that these processes elaborate and differentiate in brain evolution, and yet we know very little about the molecular regulation of the primate medial PFC, including the subgenual cingulate BA25 that is powerfully positioned to activate the stress response. Future research may find distinct molecular regulation of PFC subcircuits that may help us target therapies more effectively.
